# A Balanced Act: The Effects of GH–GHR–IGF1 Axis on Mitochondrial Function

**DOI:** 10.3389/fcell.2021.630248

**Published:** 2021-03-18

**Authors:** Bowen Hu, Hongmei Li, Xiquan Zhang

**Affiliations:** ^1^State Key Laboratory for Conservation and Utilization of Subtropical Agro-Bioresources, South China Agricultural University, Guangzhou, China; ^2^Guangdong Provincial Key Lab of AgroAnimal Genomics and Molecular Breeding, Key Lab of Chicken Genetics, Breeding and Reproduction, Ministry of Agriculture, Guangzhou, China

**Keywords:** growth hormone, growth hormone receptor, insulin-like growth factor 1, mitochondrial biogenesis, mitophagy, mitochondrial function

## Abstract

Mitochondrial function is multifaceted in response to cellular energy homeostasis and metabolism, with the generation of adenosine triphosphate (ATP) through the oxidative phosphorylation (OXPHOS) being one of their main functions. Selective elimination of mitochondria by mitophagy, in conjunction with mitochondrial biogenesis, regulates mitochondrial function that is required to meet metabolic demand or stress response. Growth hormone (GH) binds to the GH receptor (GHR) and induces the JAK2/STAT5 pathway to activate the synthesis of insulin-like growth factor 1 (IGF1). The GH–GHR–IGF1 axis has been recognized to play significant roles in somatic growth, including cell proliferation, differentiation, division, and survival. In this review, we describe recent discoveries providing evidence for the contribution of the GH–GHR–IGF1 axis on mitochondrial biogenesis, mitophagy (or autophagy), and mitochondrial function under multiple physiological conditions. This may further improve our understanding of the effects of the GH–GHR–IGF1 axis on mitochondrial function, which may be controlled by the delicate balance between mitochondrial biogenesis and mitophagy. Specifically, we also highlight the challenges that remain in this field.

## Introduction

Growth hormone (GH), also known as somatotropin, is an amino acid peptide that, together with prolactin (PRL) and human placental lactogen, belongs to the somatotropin family (Strobl and Thomas, 1994). GH is produced by the pituitary gland, which is under the positive control of the hypothalamic peptide GH-releasing hormone (GHRH) and the negative feedback of somatostatin. GH mediates its functions directly through its receptor (GHR) or indirectly via insulin-like growth factor 1 (IGF1) (Yang et al., 2008). Meanwhile, a complex feedback system can regulate GH secretion, including IGF-1, leptin, and ghrelin along with the central nervous system.

GH receptor, a member of the class I cytokine receptor family, is an amino acid dimeric receptor with an extracellular domain (ECD), a single-pass transmembrane domain (IMD), and a cytoplasmic intracellular domain (ICD) (Dehkhoda et al., 2018). *GHR* is widely expressed in GH target cells, which can combine with GH to activate diverse signal cascades, including mitogenic signaling through Janus kinase (JAKs) signal transducers and activators of transcription (STATs) (Brooks et al., 2008), mitogen-activated protein kinase (MAPK) (Vanderkuur et al., 1997), phosphoinositide-3-kinase (PI3K)/Protein kinase B (PKB or AKT)/mammalian target of rapamycin (mTOR) pathways (Hayashi and Proud, 2007) and phospholipase C (PLC)/Protein kinase C (PKC) (Bergan et al., 2015). Accordingly, normal function of GHR directly influences the physiological effects of GH. The deficiency in *GHR* will further disrupt the normal development of the organism and lead to dwarfism phenotype in a wide array of species (Lin et al., 2018).

Insulin-like growth factor 1, the main mediator of GH actions, is an amino acid insulinoid peptide with an amino acid sequence similar to that of proinsulin (positions 1–29 are homologous to insulin B chain and positions 42–62 to insulin A chain). GH combines with GHR to regulate IGF1 production via the JAK2/STAT5 pathway through endocrine and paracrine/autocrine mechanisms (Sjogren et al., 1999; Junnila et al., 2013). IGF1 mainly occurred in the liver and also in several tissues including the brain, testes, skeletal muscle, bone, and cartilage. Meanwhile, the roles of GH and IGF1 are influenced by GH-binding proteins (GHBPs) and IGF-binding proteins (IGFBPs), respectively (Baumann et al., 1988; Duan and Xu, 2005). Analogous to GH, IGF1 acts through its receptor (IGF1R), a tyrosine kinase receptor that can activate multiple pathways including the PI3K/AKT, MAPK, and PLC pathways (Hakuno and Takahashi, 2018). However, GH and IGF-1 have very different roles on glucose and lipid metabolism. GH primarily blocks the action of insulin, promotes lipolysis, and prevents fat production, whereas IGF1 has the opposite effects (Moller and Jorgensen, 2009).

**FIGURE 1:**
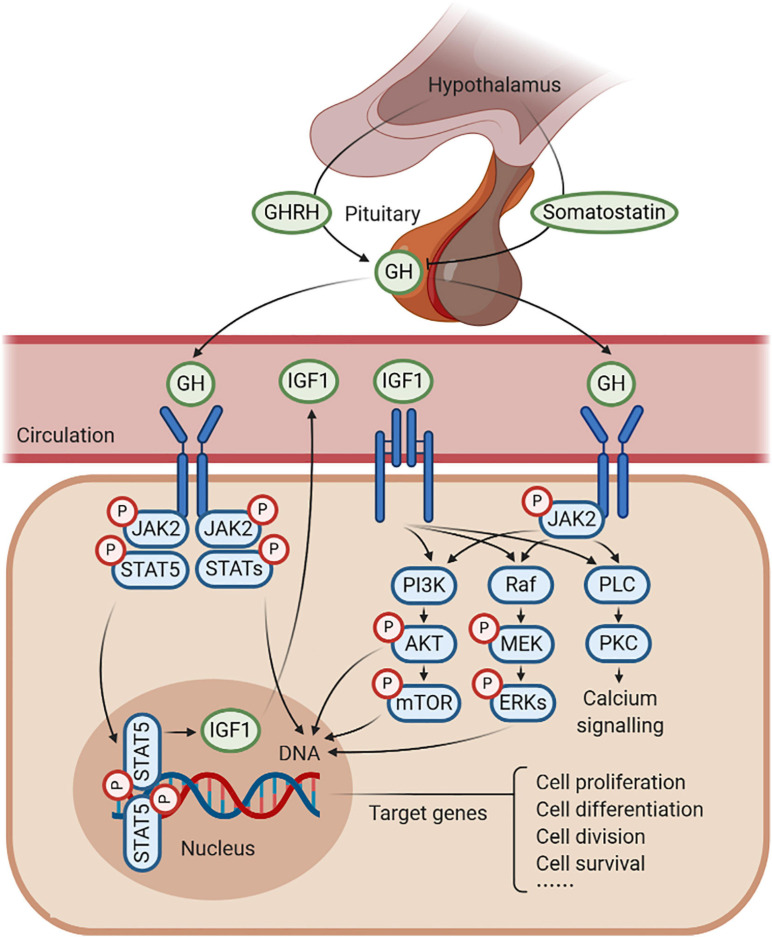
Schematic representation of growth hormone (GH)–GH receptor (GHR)–insulin-like growth factor 1 (IGF1) axis. GH is produced by the pituitary gland, which is under the positive control of the GH-releasing hormone (GHRH) and the negative feedback of somatostatin. GH combines with GHR to regulate the production of IGF1, in response to somatic growth, including cell proliferation, cell differentiation, cell division, and cell survival. In this process, several signaling pathways are activated, such as JAK2/STAT5, MAPK, and PI3K/AKT signaling under an anabolic state (such as during periods of feeding), as well as PLC/PKC signaling under a catabolic state (such as during periods of fasting).

Overall, the GH–GHR–IGF1 axis, part of the somatotropic–hypothalamic–pituitary axis, has been commonly recognized in response to somatic growth, including cell proliferation, differentiation, division, and survival (Figure 1). On the other hand, the GH–GHR–IGF1 axis also plays essential roles in mitochondrial function with an unexpected complexity and versatility regulation mechanisms. In this review, we describe recent discoveries providing evidence for the contribution of the GH–GHR–IGF1 axis on mitochondrial biogenesis, mitophagy (or autophagy), and mitochondrial function under multiple physiological conditions. Based on this integrative view, we also emphasize the remaining challenges in this field. Besides, there is a long list of studies utilizing different cell lines or mice with varying membership of the GH–GHR–IGF1 axis, showing the effects of the GH–GHR–IGF1 axis on aging and cellular senescence. Although important, they were not described primarily in this review.

## Overview of the Mitochondrial Biogenesis

Mitochondria are double membrane-bound organelles, especially with the generation of adenosine triphosphate (ATP) through oxidative phosphorylation (OXPHOS) being one of their main functions. The OXPHOS proteins comprise five complexes (I–V) embedded in the inner mitochondrial membrane that are uniquely controlled by mitochondrial DNA (mtDNA) and the nuclear DNA (nDNA). In mammals, the mtDNA encodes only 37 genes, of which 13 are subunits of the OXPHOS (complexes I, III, IV, and V), two are rRNA genes, and 22 are tRNA genes required for mitochondrial protein synthesis.

Mitochondrial biogenesis is a self-renewal process by which new mitochondria are produced from the ones already existing. The sophisticated process of mitochondrial biogenesis requires coordination between mtDNA and nDNA, including mtDNA transcription and translation, translation of nDNA-encoded transcripts, membrane recruitment, protein import, and assembly of the OXPHOS complexes (Attardi and Schatz, 1988). So far, it has been widely accepted that the members of the peroxisome proliferative-activated receptor gamma coactivator 1 (PGC1) family of coactivators act as key players in the regulation of energy metabolism and mitochondrial biogenesis. PPARG coactivator 1 alpha (PGC1α) was first described in 1999 (Wu et al., 1999), and PPARG coactivator 1 beta (PGC1β) along with PPARG related-coactivator 1 (PRC) were discovered subsequently (Andersson and Scarpulla, 2001; Lin et al., 2002). These coactivators generally orchestrate with DNA-bound transcription factors, such as nuclear respiratory factors (NRF1, NRF2), estrogen-related receptors (ERRα, ERRβ, and ERRγ), and myocyte enhancer factor 2 (MEF2) to drive the expression of target genes. Furthermore, their regulations on transcription or post-translation level are responsive to meet the multiple metabolic demands induced by physiological signals, senescence, and diseases (Cui et al., 2006; Bellance et al., 2009; D’Errico et al., 2011; Patel et al., 2012; Tsunemi and La Spada, 2012). For instance, PGC1-related coactivators can activate the expression of NRF1, NRF2, and transcription factor A (TFAM, the final effectors of mtDNA transcription and replication) to regulate the expression of respiratory chain and the biogenesis of mitochondria, a process that has been well-documented in previous reviews (Gleyzer et al., 2005; Scarpulla, 2008; Scarpulla et al., 2012; Villena, 2015; Popov, 2020). Therefore, we commonly regard the expression of *PGC1*α, *NRF1*, *TFAM*, and mitochondrial-related genes, as well as mtDNA copy number as the markers for mitochondrial biogenesis. Notably, a recent study has underlined that the relationship between *TFAM* expression and mitochondrial biogenesis is more complex than is generally appreciated and may be ambiguous in most mammalian cells. As TFAM does not always exhibit parallel with the mtDNA copy number, TFAM should be used judiciously as a marker of mitochondrial biogenesis (Kozhukhar and Alexeyev, 2019).

## Overview of the Mitophagy

Autophagy, meaning “self-eating” in Greek, is a process by which portions of cytoplasm, such as the organelles and protein aggregates, are sequestered and subsequently delivered to lysosomes for degradation (Klionsky et al., 2003; Nakatogawa et al., 2009). Three major types of autophagic pathways, macroautophagy, microautophagy, and chaperone-mediated autophagy (CMA), have been recognized in eukaryotic cells (Mizushima and Komatsu, 2011). Macroautophagy (hereafter referred to as autophagy) sequesters cytosolic cargo, mainly organelles, by a double membrane vesicle called autophagosome, which is formed through conjugation of specific proteins among themselves in an intricate process. Subsequently, autophagosome is fused with endolysosome to constitute autolysosome, in which the cytosolic cargo is degraded. Although autophagy was first considered to perform in a bulk manner, it is now clear that autophagy is mainly a selective process that originally encounters considerable resistance (Mizumura et al., 2014).

Early in the 20th century, the concept of mitochondrial degradation was proposed by Margaret and Warren Lewis (Lewis and Lewis, 1915). Later, mitochondria sequestered in lysosomes were first observed in rat tissues by electron microscopy studies (Ashford and Porter, 1962; Novikoff and Essner, 1962). Until the beginning of the 21st century, the idea of mitochondrial autophagy was initially termed as “mitophagy” to define the selective elimination of depolarized mitochondria in autophagosome (Scott and Klionsky, 1998; Elmore et al., 2001; Lemasters, 2005). Up to now, mitophagy is considered as a selective form of autophagy to deliver dysfunctional or superfluous mitochondria to the lysosome for degradation, which has exhibited an essential contribution to cell homeostasis under different stimuli or cellular contexts, including cellular differentiation, oxidative stress response, and aging, as well as various disease conditions (Um and Yun, 2017; Palikaras et al., 2018). In yeast, Ohsumi’s and Klionsky’s laboratories first identified that ATG32 acts as the mitophagy-specific receptor, which directly interacts with the selective autophagy adaptor ATG8 via an ATG8-interacting motif (AIM) and ATG11 to form the nascent autophagosome (Kanki et al., 2009; Okamoto et al., 2009). Recently, ATG43 is identified as another mitophagy-specific receptor, which is localized on the mitochondrial outer membrane and binds to ATG8 (Fukuda and Kanki, 2021).

In mammals, the regulation of mitophagy appears to be more complex, both ubiquitin-mediated and receptor-mediated pathways have been described in response to mitophagy. To our knowledge, the most studied and understood mitophagy pathway is mediated by PTEN-induced kinase 1 (PINK1) and the E3 ubiquitin ligase Parkin (Narendra et al., 2008; Vives-Bauza et al., 2010), both of which have been linked to forms of Parkinson’s disease (Kitada et al., 1998; Valente et al., 2004). The complicated mechanisms of canonical and non-canonical PINK1/parkin-mediated mitophagy have been well summarized in previous reviews (Eiyama and Okamoto, 2015; Lazarou et al., 2015; Nguyen et al., 2016; Clark et al., 2020; Malpartida et al., 2020). Moreover, two main types of receptor-mediated mitophagy pathway have been classified as follows in brief: ubiquitin-independent mitophagy receptors, including BCL2-interacting protein 3 (BNIP3) (Quinsay et al., 2010), BCL2-interacting protein 3 like (NIX/BNIP3L) (Sandoval et al., 2008), FUN14 domain-containing 1 (FUNDC1) (Liu et al., 2012), BCL2-like 13 (BCL2L13) (Otsu et al., 2015), autophagy and beclin 1 regulator 1 (AMBRA1) (Strappazzon et al., 2015), FKBP prolyl isomerase 8 (FKBP8) (Bhujabal et al., 2017), prohibitin 2 (PHB2) (Wei et al., 2017), and NLR family member X1 (NLRX1) (Zhang et al., 2019); lipid-mediated mitophagy receptors, including ceramide (Sentelle et al., 2012) and cardiolipin (Li et al., 2015). It has been identified that these mitophagy receptors can directly interact with the autophagy mediators LC3/GABARAP via a conserved LC3-interacting region (LIR) motif or ULK1 protein to form the nascent autophagosome. These intricate processes of receptor-mediated mitophagy pathways also have been well summarized in previous reviews (Liu et al., 2014; Ploumi et al., 2017; Villa et al., 2018; Montava-Garriga and Ganley, 2020). However, whether each type of mitophagy receptor functions in a distinct pathway, or there is cooperation between them under various mitochondrial stresses are still not completely understood.

## A Balanced Act of Mitochondrial Biogenesis and Mitophagy

Mitochondrial function is multifaceted in response to cellular energy homeostasis and metabolism, including calcium homeostasis, amino acid metabolism, pyridine synthesis, cellular replication, apoptosis, reactive oxygen species (ROS) production, and senescence (Spinelli and Haigis, 2018). In order to perform these many functions, mitochondria are structured in a dynamic network where, for instance, mitochondria biogenesis, elimination, fission, and fusion are harmoniously orchestrated (Ploumi et al., 2017). Accordingly, maintenance of a healthy mitochondrial network, defined as mitochondrial homeostasis, is critical for normal mitochondrial function during development and even throughout life. Like ancient Chinese philosophy “Ying” and “Yang,” both generation of newly synthesized mitochondria, by mitochondrial biogenesis, and elimination of detrimental and/or superfluous mitochondria, by mitophagy, are predominantly required for maintaining mitochondrial homeostasis. Recent findings have hinted that any abnormality in these two opposing processes can influence the quantity and quality of mitochondria, which will further affect mitochondrial function and the ability of cells to adjust their mitochondrial networks in response to physiological adaptations and stress conditions (Palikaras et al., 2015; Singh et al., 2018; Wang et al., 2019; Yau et al., 2019; Zhou et al., 2019; Chen et al., 2020). At the same time, impaired mitochondrial function and homeostasis are now widely accepted to be associated with multiple aspects of the aging process and age-onset diseases (Lopez-Otin et al., 2013; Mattson and Arumugam, 2018; Akbari et al., 2019).

**FIGURE 2:**
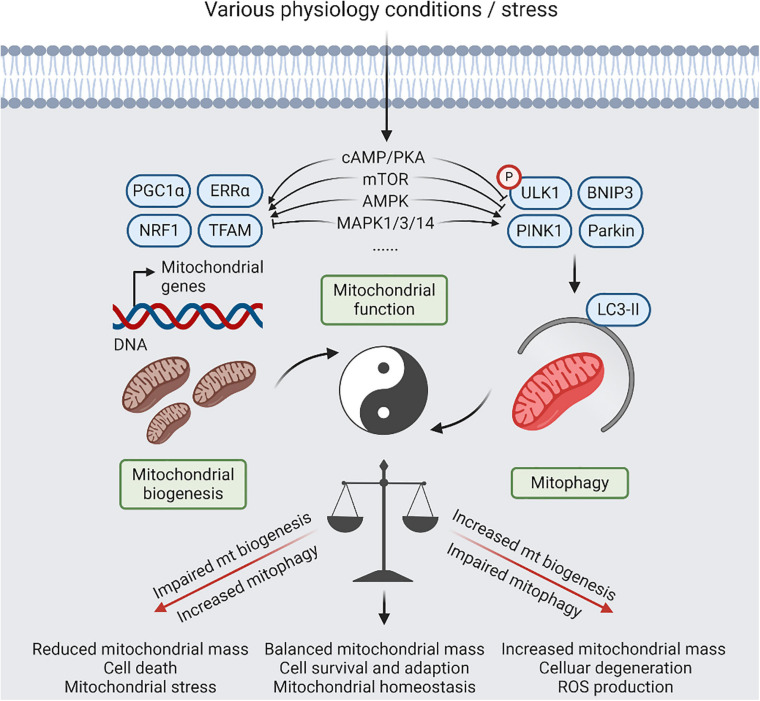
A balanced act of mitophagy and mitochondrial biogenesis. Coordination between mitochondrial (mt) biogenesis and mitophagy results in generation of new synthesized mitochondria, and elimination of detrimental and/or superfluous mitochondria, which is controlled by several signaling pathways, including cyclic-AMP (cAMP)/protein kinase A (PKA), AMP-activated protein kinase (AMPK), mitogen-activated protein kinase (MAPK), and mammalian target of rapamycin (mTOR) signaling, in response to physiological adaptations and stress conditions. Relative to normal condition, increased mitophagy or impaired mt biogenesis will lead to reduced mitochondria mass, contributing to cell death that be dependent on mitochondrial function. Reversely, impaired mitophagy or increased mt biogenesis will lead to imbalanced responses, resulting in increased mitochondria mass, increased reactive oxygen species (ROS) production, and cellular degeneration. However, restoration of mitochondrial homeostasis with increased mitochondrial damage will require simultaneous upregulation of mitophagy and mitochondrial biogenesis.

Therefore, maintaining the healthy function of mitochondria by biogenesis and mitophagy is conducive to cellular life activity. The balance between mitochondrial biogenesis and mitophagy requires delicate regulation to maintain a sustainable mitochondria population in healthy cells (Pickles et al., 2018). Several signaling pathways have been implicated in both mitochondrial biogenesis and mitophagy, and they may play important roles in coordinating these processes (Figure 2). For instance, cyclic-AMP (cAMP) is one of the upstream signals that regulate both mitochondrial biogenesis and mitophagy. cAMP level can regulate the protein kinase A (PKA)-dependent activation of the cAMP response element-binding protein (CREB), which in turn upregulates the expression of PGC1α and inhibits LC3-II (Cherra et al., 2010; Chowanadisai et al., 2010). Also, mammalian target of rapamycin (mTOR) signaling promotes mitochondrial biogenesis by activation of PGC1α and ERRα, and inhibits mitophagy by phosphorylation of ULK1 or inhibition of PINK1 expression and Parkin translocation (Cunningham et al., 2007; Bartolome et al., 2017). Under energy stress condition, AMP-activated protein kinase (AMPK) promotes mitochondrial biogenesis through phosphorylation of SIRT1 to activate PGC1α, and promotes mitophagy through inhibition of mTOR and activation of ULK1 (Herzig and Shaw, 2018). Also, mitogen-activated protein kinase (MAPK) signaling is associated with mitochondrial homeostasis, MAPK1/3 potently inhibit mitochondrial biogenesis (Zhu et al., 2012); however, p38 MAPK exerts a positive regulation on PGC1α, and MAPK1 along with MAPK14 promote both starvation- and hypoxia-induced mitophagy in HeLa cells (Akimoto et al., 2005; Hirota et al., 2015).

On the other hand, in addition to its well-known functions, PGC1α, a master regulator of mitochondrial biogenesis, positively regulates mitophagy by directly inducing the expression of transcription factor EB (TFEB) that mediates lysosomal biogenesis (Settembre et al., 2011). NRF2 is also required to couple mitochondrial biogenesis with mitophagy by inducing *BNIP3* expression, which is essential for facilitating cancer progression (Riis et al., 2020). Meanwhile, Parkin is involved in the regulation of mitochondrial biogenesis via PARIS, a transcription factor that negatively regulates PGC1a and its target NRF1 or directly enhances TFAM-mediated mitochondrial transcription in proliferating cells (Kuroda et al., 2006; Shin et al., 2011). Furthermore, it was found that the general control of amino acid synthesis 5-like 1 (GCN5L1) negatively regulates both mitochondrial biogenesis and degradation pathways through acting on both PGC1a and TFEB in mouse embryonic fibroblasts (Scott et al., 2014).

So far, many upstream signals have been implicated in both mitochondrial biogenesis and mitophagy as described above. However, there is still a lack of systematic research on whether there are interactions among them. Notably, the versatility of the key regulators of mitochondrial biogenesis (PGC1α, NRF2) and mitophagy (Parkin) may further underline that these two processes are balanced and constrained with each other in order to control mitochondrial function under multiple physiological conditions.

## The Effects of Growth Hormone on Mitochondrial Biogenesis

The relationship between GH and mitochondrial biogenesis was initially explored in the early 1970s. The first question is whether the GH performs a direct interaction with mitochondrial membranes (Maddaiah et al., 1970). After administration of radio-labeled bovine GH to rats, radioactive signals were detected in the mitochondria of liver and kidney (Groves et al., 1972). Later, this question was further explored by Mutvei et al. in hypophysectomized rats treated with T3 and/or human GH by the continuous infusion of hormone for 6 days. Intriguingly, they found that T3 exerts a direct effect on mitochondrial biogenesis. However, the high-affinity binding sites for GH are not present in the liver mitochondria; only a few negligible amounts of radio-labeled bovine GH are transported to the mitochondria compared with other subcellular compartments (Mutvei et al., 1989). This compelling evidence demonstrates that mitochondria are not a direct target for GH and/or its receptor.

Growth hormone is considered to be a conducive hormone that enhances mitochondrial biogenesis. Human GH treatment of hypophysectomized rats increases incorporation of leucine *in vivo* and *in vitro*, indicating that GH has a significant effect on liver mitochondrial protein synthesis (Maddaiah et al., 1973). Administration of either human or bovine GH restores the cytochrome level and increases the cytochrome oxidase activity in hypophysectomized rats (Maddaiah et al., 1976). Also, administration of bovine GH to hypophysectomized rats partially restores the respiration rate and ATPase activity of liver mitochondria, and increases the heart mitochondrial protein synthesis measured by the incorporation capacity of radioactive leucine (Katkocin et al., 1979; Maddaiah and Clejan, 1986). Furthermore, GH replacement therapy restores the age-associated impairments in the skeletal muscle mitochondrial biogenesis, which is mainly manifested by the increased *PGC1*α, *NRF1*, *cytochrome c* expression, and citrate synthase enzymatic activity (Brioche et al., 2014). Consistently, PGC1α protein level is diminished in bovine GH transgenic (bGH Tg) mice, which overexpresses GH and are short-lived (Al-Regaiey et al., 2005). These investigations are consistent with the results in the early 1970s showing the beneficial effects of GH on mitochondrial biogenesis.

On the other hand, some different views concerning the effects of GH on mitochondrial biogenesis have also been discovered. The expression of *PGC1*α and OXPHOS activities are increased in the long-living Ames dwarf mice (lack of GH, prolactin, and thyroid-stimulating hormone), demonstrating a negative effect of GH on mitochondrial biogenesis (Westbrook et al., 2009; Brown-Borg et al., 2012). Moreover, Mutvei et al. (1989) suggested that GH is not a major regulator of mammalian mitochondrial biogenesis. They considered that previous results concerning the effect of GH on mitochondrial biogenesis were based on the incorrect interpretations of the data, as increased respiration does not necessarily reflect increased mitochondrial biogenesis. Similarly, it was recently reported that the expression of mitochondria-specific markers (*PGC1*α, *AMPK*α, *SIRT1*, and *cytochrome b*, etc.) along with the protein levels of electron transport chain (complexes I, II, III, IV, and V) in osteocytes are not significant differences between bGH Tg and control mice (Liu et al., 2019). Taken together, these different results mentioned above indicate that GH may account for complex functions on mitochondrial biogenesis during different physiological conditions *in vivo* and *in vitro*.

## The Effects of Growth Hormone on Mitophagy

Mitophagy is a well-studied type of cargo-specific autophagy to selectively eliminate mitochondria. However, no direct relationship between GH and mitophagy has been reported. Therefore, in this section, we only summarize the connection between GH and autophagy, which may throw the rope for the future researches.

Early study initially suggested that disruptions of the insulin or GH/IGF1 axis with low insulin and IGF1 levels may enhance autophagy to prevent the age-related mitochondrial degradation and extend the lifespan (Bergamini et al., 2003). Wang and Miller (2012) demonstrated that fibroblasts in Snell mice (which secrete very low amounts of GH, prolactin, and thyroid-stimulating hormone) are more susceptible to autophagy induced by amino acid withdrawal or by oxidative stress than control cells. They also found evidence of reduced mTOR function in dwarf cells under autophagy conditions, which is consistent with the evidence that increased autophagy requires lower mTOR activity (Wang and Miller, 2012). Furthermore, somatostatin analog treatment might induce apoptosis, increase autophagy, and decrease cell proliferation in GH-secreting adenomas (Dagistanli et al., 2018). A recent study also showed that reduced GH signaling in the liver of Snell mice upregulates the CMA (Endicott et al., 2020). These findings indicate a negative regulatory effect of GH on autophagy.

In contrast with these results, lack of the GH secretagogue ghrelin causes lethal hypoglycemia in mice under fasted and fat-depleted state; however, the wild-type mice under the same conditions exhibit a massive increase in plasma GH and hepatic autophagy, suggesting the positive connection between the plasma GH and hepatic autophagy (Zhang et al., 2015). Besides, GH acts through its receptor GHR in the liver to activate autophagy, preserve triglycerides, enhance gluconeogenesis, and prevent hypoglycemia in calorie-restricted mice (Fang et al., 2019). Accordingly, similar to the effects of GH on mitochondrial biogenesis, the findings above indicate the different roles of GH on autophagy during diverse physiological conditions *in vivo*.

## The Effects of Growth Hormone Receptor on Mitochondrial Biogenesis

In recent decades, genetically engineered mouse strains (*GHR* gene disrupted or knockout mouse) have become vital tools for exploring the various activities of GH and GHR *in vivo* (List et al., 2020). Similarly, the genetic background of the sex-linked dwarf (SLD) chickens, which are caused by *GHR* gene mutations, also allows this strain to become a model system to understand the roles of *GHR in vivo* (Luo et al., 2016).

Early in the 21st century, GHR is considered to exhibit a negative effect on the process of mitochondrial biogenesis *in vivo*. Al-Regaiey et al. (2005) published a paper in which they described that PGC1α protein level was increased in the liver of male GHRKO mice. The gene expression and protein level of mitochondrial biogenesis markers, including *PGC1*α, *AMPK*, *SIRT3*, *eNOS*, and *MFN2*, are increased in GHRKO mice (Gesing et al., 2011a, b). A recent study also showed that the PGC1α protein levels in liver are significantly increased in both male and female GHRKO pigs compared with sex-matched controls (Riedel et al., 2020). However, the decrease in *NRF1* and *TFAM* expression in the skeletal muscles and *TFAM* expression in kidneys of GHRKO mice was also demonstrated (Gesing et al., 2011b). The authors suggested that the decrease in *TFAM* may reflect potentially unaltered mtDNA content in GHRKO mice (Gesing et al., 2013). Notably, the increased expression of key regulators of mitochondrial biogenesis in GHRKO mice is not improved further by calorie restriction or visceral fat removal (Gesing et al., 2011b).

However, several recent findings are different from the previous results. Our previous study has revealed that the gene expression of mitochondrial biogenesis markers (*PGC1*α, *NRF1*, and *TFAM*) and mtDNA-encoded OXPHOS genes are all downregulated in the skeletal muscle of SLD chickens and *GHR* knockdown cells (Hu et al., 2019). At the same time, we observed that the enzymatic activities of OXPHOS complexes (complexes I, II, III, and IV) are reduced in the skeletal muscle of SLD chickens and *GHR* knockdown cells, indicating that GHR exhibits a positive effect on mitochondrial biogenesis. Furthermore, the expression of mitochondria-specific markers (*PGC1*α, *AMPK*α, *SIRT1*, and *cytochrome b*, etc.) and the protein levels of OXPHOS complexes (complexes I, II, III, IV, and V) in osteocytes are not significantly different between GHRKO and control mice (Liu et al., 2019). This investigation suggested that GHR has no effect on mitochondrial biogenesis, at least *in vitro*. On the other hand, the gene expression of mitochondrial biogenesis markers (*PGC1*α, *AMPK*, *SIRT1*, *NRF2*, and *MFN2*) and mitochondrial activity marker (*COXIV*) in liver-specific GHRKO (LiGHRKO) and wild-type mice are significantly different between the males and females, suggesting that sexual dimorphism may also play an essential role in regulating the mitochondrial biogenesis (Zawada et al., 2015).

As the middle of the GH–GHR–IGF1 axis, GHR plays a pivotal role in its functions. Taking into account these important observations, it seems that the effects of GH and GHR on mitochondria biogenesis are similar, as they both exhibit a multifaceted feature that are summarized in Table 1. There may be many explanations accounting for this difference. One explanation might be that the roles of GH and GHR on mitochondrial biogenesis may be different among cell-, organ-, and species-specific factors. The other explanation might be that mitochondrial biogenesis *per se* is not always assayed. The induction or repression of some mitochondrial markers is not always representative of the expansion of the mitochondrial network.

**TABLE 1:** The multiple effects of growth hormone (GH) and growth hormone receptor (GHR) on mitochondrial biogenesis.

Protein	Impact on mitochondrial biogenesis	Model	Tissue or cell	References
GH	Positive effect	Hypophysectomized rats	Liver, heart	Maddaiah et al., 1973, 1976; Katkocin et al., 1979; Maddaiah and Clejan, 1986
		Bovine GH transgenic mice	Liver	Al-Regaiey et al., 2005
		Rats	Skeletal muscle	Brioche et al., 2014
	Negative effect	Long-living Ames dwarf mice	Liver	Westbrook et al., 2009; Brown-Borg et al., 2012
	No effect	Hypophysectomized rats	Liver	Mutvei et al., 1989
		Bovine GH transgenic mice	Osteocyte	Liu et al., 2019
GHR	Positive effect	GHRKO mice	Liver	Al-Regaiey et al., 2005
		GHRKO mice	Skeletal muscle, kidney	Gesing et al., 2011a, b
		GHRKO pigs	Liver	Riedel et al., 2020
	Negative effect	SLD chickens	Skeletal muscle, DF-1 cell	Hu et al., 2019
	No effect	GHRKO mice	Osteocyte	Liu et al., 2019

## The Effects of Growth Hormone Receptor on Mitophagy

In recent decades, similar to GH, no direct relationship has been reported between GHR and mitophagy. Therefore, in this part, we merely summarized the connection between GHR and autophagy from sporadic studies. It was found that *GHR* expression and its protein level are reduced in the skeletal muscle of ATG7 knockout mice, implying a synchronous relationship between GHR and autophagy (Zecchini et al., 2019). Furthermore, the level of the autophagy marker LC3-II is increased in GHRKO osteocytes (Liu et al., 2019). A recent study also showed that LC3-II flux is increased in the liver of GHRKO mice, but unaltered in LiGHRKO mice (Endicott et al., 2020). Up to now, the above two studies suggest a negative regulatory effect of GHR on autophagy. These new findings only reveal a fraction of the relationship between GHR and autophagy. Meanwhile, it is currently observed that these multifunctional effects of GHR on autophagy may be similar to GH. In the future, more research will be needed to deepen our understanding of the relationship between GHR and autophagy, even mitophagy.

## The Effects Of Insulin-Like Growth Factor 1 on Mitochondrial Biogenesis

Cells generate new mitochondria when stimulated by extracellular factors to grow and divide. Numerous studies have aimed at assessing the effects of IGF1 on mitochondrial biogenesis. Neuregulin and IGF1 can act synergistically to increase mitochondrial biogenesis and mtDNA replication in primary Schwann cells, which requires both the ERK and PI3K signaling pathways (Echave et al., 2009). This process is mediated by the transcription factor ERRα and is independent of AKT/mTOR signaling pathways. IGF1 also enhances the level of mitochondrial protein involved in signal transduction, protein import and folding, mtDNA transcription, and bioenergetics in Huntington’s disease (HD) striatal cells (Ribeiro et al., 2014). Similarly, the aging rats untreated with IGF1 exhibit a significant mitochondrial dysfunction, including reduced activity of ATPase and complex IV (Garcia-Fernandez et al., 2008; Puche et al., 2008). Furthermore, cMYC regulates the expression of *PGC1*β in breast cancer cells in response to Her2/IGF1 activation (Chang et al., 2011). There is also evidence showing that IGF1 promotes mitochondrial biogenesis through the induction of PGC1β and PRC, not PGC1α, *in vitro* (Lyons et al., 2017). Likewise, loss of IGF1 signal reduces the expression of mitochondrial biogenesis markers (*PGC1*α, *TFAM*) in the steroidogenic cells of prepubertal testis (Radovic et al., 2019). Overall, these compelling evidences demonstrate that IGF1 acts as a protector in the process of regulating mitochondrial biogenesis.

## The Effects of Insulin-Like Growth Factor 1 on Mitophagy

Up to now, numerous studies have reported the relationship between IGF1 and autophagy. Here, we only briefly elucidate as follows. IGF1 inhibits starvation-induced cardiac autophagy via mTOR signaling *in vitro*, and negatively regulates cardiac autophagy and AMPK activity *in vivo* (Troncoso et al., 2012). Conversely, high protein levels of IGF1 and its receptors, accompanied by a reduction in AKT/mTOR signaling pathways resulting from resistance exercise training, are associated with increased autophagy activity in aged skeletal muscles (Luo et al., 2013). Also, *IGF1* expression is significantly reduced in ATG7 knockout mice, indicating that IGF1 plays a beneficial role in regulating autophagy (Zecchini et al., 2019).

Nevertheless, the role of IGF1 on mitophagy is rarely reported. IGF1 can induce the expression and accumulation of BNIP3 in mitochondria through a PI3K-dependent manner, indicating that IGF1 promotes mitophagy *in vitro* (Lyons et al., 2017). In mouse and cell models of amyotrophic lateral sclerosis (ALS), IGF1 also strongly protects mitochondria from apoptosis and upregulates mitophagy, as evidenced by a decrease in the p62 level and an increase in the LC3-II level (Wen et al., 2019). Recently, a study further revealed that IGF1-induced *BNIP3* expression requires NRF2 to act through downstream transcriptional factors HIF-1α and NRF1 (Riis et al., 2020). These novel findings above strongly demonstrate that IGF1 promotes the process of mitophagy both *in vivo* and *in vitro*. However, whether there is an interaction between the mitochondrial biogenesis and mitophagy regulated by IGF1 is still unknown, and further research is needed.

## The Effects of GH–GHR–IGF1 Axis on Mitochondrial Function

Many methods have been utilized to measure normal mitochondrial function or dysfunction in different systems. Generally, mitochondrial respiration control, including oxygen consumption rate (OCR) and respiratory control ratio (RCR), is utilized to measure the mitochondrial function in diverse cell populations (Brand and Nicholls, 2011). Mitochondrial membrane potential (ΔΨm) is also used as an indicator for mitochondrial function. Loss of ΔΨm normally indicates mitochondrial dysfunction and is accompanied by increased mitochondrial swelling (Javadov et al., 2006, 2009). Furthermore, reduced ΔΨm will lead to uncoupling of OXPHOS and increases ROS production accompanied by elevated malondialdehyde (MDA) levels and reduced ATP levels (Lebiedzinska et al., 2010; Bai et al., 2017). Notably, numerous studies have revealed a positive correlation between ΔΨm and ROS production in various physiological and pathological scenarios. Mitochondrial proton leakage, mainly due to the decrease in ΔΨm, is considered to counteract mitochondrial ROS production to protect cells from oxidative stress (Turrens, 2003; Mailloux and Harper, 2011).

Until now, a long list of studies utilizing numerous cell types has shown the effects of the GH–GHR–IGF1 axis on mitochondrial function *in vivo* and *in vitro*. Early in the 1970s, most of researches had revealed the positive effects of GH on mitochondrial respiration rate and its related enzyme activities in hypophysectomized rats (Maddaiah et al., 1976; Katkocin et al., 1979; Maddaiah and Clejan, 1986). Short-term GH therapy for 3 months increases the activity of succinate dehydrogenase, which represents the mitochondrial function in human quadriceps muscle (Gonzalez et al., 2018). A recent study also demonstrated that the OCR and ATP production is significantly increased in primary osteocytes of bGH Tg mice (Liu et al., 2019). These results suggested that GH has a positive effect on mitochondrial function *in vivo*. Furthermore, increased OXPHOS activities and oxygen consumption, along with reduced ROS production were found in the long-living Ames dwarf mice (Westbrook et al., 2009). Also, decreased oxygen consumption was observed in bGH Tg mice (Brown-Borg et al., 2012). These findings indicated that GH has a negative effect on mitochondrial function *in vivo*. In addition, administration of GH by the continuous infusion of hormone for 6 days in hypophysectomized rat liver has no effect on mitochondrial respiration and enzyme activities (Mutvei et al., 1989).

These discrepancy results can also be observed on GHR functions. *GHR* ablation is detrimental to osteocyte and fibroblast mitochondrial function (Liu et al., 2019). Consistently, mitochondrial function is impeded in the skeletal muscle of SLD chickens and *GHR* knockdown cells, indicating that GHR promotes mitochondrial function *in vivo* and *in vitro* (Hu et al., 2019). However, these data are in conflict with a previous report that demonstrated an enhanced mitochondrial function in the liver, muscle, heart, kidneys, and brain of aged GHRKO mice (Brown-Borg et al., 2009). Also, the abundance of three tricarboxylic acid cycle (TCA) cycle enzymes (isocitrate dehydrogenase, fumarase, and malate dehydrogenase) is significantly increased in the GHRKO pigs’ liver proteome, suggesting that GHR inhibits mitochondrial function *in vivo* (Riedel et al., 2020). Due to the lack of GH and GHR on the regulation of mitophagy, we proposed that these discrepant results of GH and GHR on mitochondrial function may be explained by the coordinated regulation mechanism between mitochondrial biogenesis and mitophagy to meet different physiological conditions *in vivo* and *in vitro*.

In the last 10 years, most evidence has revealed that IGF1 is regarded as a protector for mitochondrial function under several diseases and stress conditions. IGF1 not only protects cardiomyocytes from hypoxia/reoxygenation injury by stabilizing ΔΨm and reducing ROS damage but also alleviates mitochondria dysfunction in cardiomyocytes from high-fat diet mice (Pi et al., 2007; Zhang et al., 2012). In neuronal cells, IGF1 protects against prion diseases caused by mitochondrial dysfunction and increased ROS production via inhibition of Bax translocation (Park et al., 2012). Consistently, IGF1 increases ΔΨm in HD striatal cells in a PI3K/AKT-dependent manner (Ribeiro et al., 2014). Activation of IGF1 signaling pathways also ameliorates O_2_ consumption and ΔΨm in HD lymphoblasts (Naia et al., 2015). Furthermore, regulation of astrocytic mitochondrial function and redox status by IGF1 is essential to maintain astrocytic function and coordinate hippocampal-dependent spatial learning (Logan et al., 2018). Knockdown of the *IGF1* leads to a reduction in ΔΨm and alterations in mitochondrial morphology in ALS mice (Wen et al., 2019). In addition, IGF1 activates AMPK to augment mitochondrial function (OCR and ATP production) in sensory neurons in type-1 diabetes (Aghanoori et al., 2019). Of note is that a recent study revealing that induced liver IGF1 knockout can impair hippocampal mitochondrial OXPHOS coupling efficiency and reduce cortex ATP levels (Pharaoh et al., 2020). However, IGF1 has no significant impact on muscle mitochondrial function, indicating that the deficiency of IGF1 in male mice has different roles on tissue mitochondrial function between the center and periphery circulation (Pharaoh et al., 2020). Taken together, these findings strongly demonstrate that IGF1 promotes mitochondrial function to restore various diseases and stress conditions, such as improving metabolism and exerting mitochondrial protection, hepatoprotective as well as neuroprotective effects.

Accordingly, Sadaba et al. (2016) suggested that one of the newest targets to recover mitochondrial dysfunction could be the administration of low doses of IGF1. This is supported by compelling evidences that IGF1 replacement therapy is able to restore mitochondrial dysfunction observed in untreated cirrhotic rats and in IGF1 partial deficiency mice (Perez et al., 2008; Olleros et al., 2017).

## Future Perspectives

**FIGURE 3:**
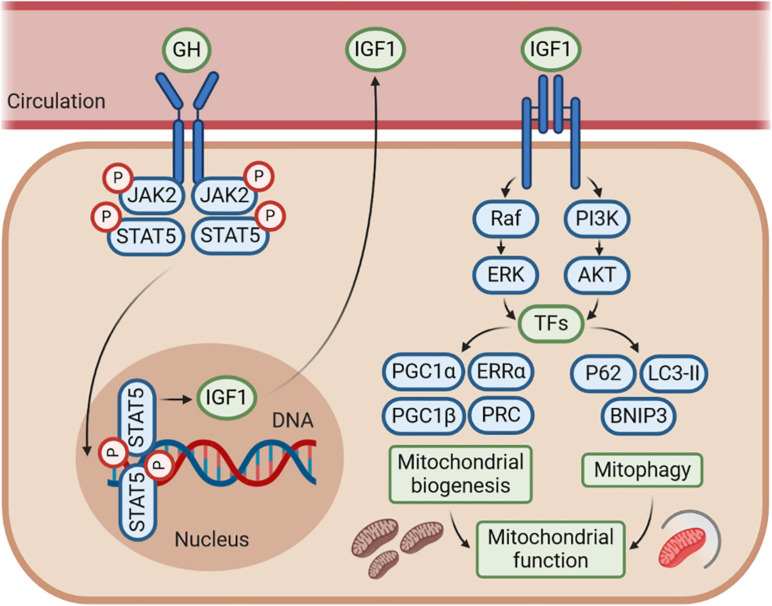
The GH–GHR–IGF1 axis may control mitochondrial function by regulating mitochondrial biogenesis and mitophagy. The effects of GH–GHR–IGF1 axis on mitochondrial function may be mostly mediated by IGF1, which stimulates several signaling pathways, including phosphoinositide-3-kinase (PI3K)/AKT and Ras/Raf/MAPK signaling, to further activate numerous transcription factors (TFs). This cascade leads to transcriptional activity of genes involved in both mitochondrial biogenesis and mitophagy. Accordingly, IGF1 is commonly considered to enhance mitochondrial function by promoting mitochondrial biogenesis and mitophagy under several conditions of metabolic or mitochondrial stress. However, whether there is an interaction between the mitochondrial biogenesis and mitophagy regulated by IGF1 is still unknown, and further research is needed.

To sum up, it is generally believed that IGF1 enhances mitochondrial function by promoting both mitochondrial biogenesis and mitophagy under several conditions of metabolic or mitochondrial stress. Accordingly, regulation of IGF1 secretion may have a therapeutic potential in the protection of mitochondrial function for treating many mitochondria-related diseases. In Figure 3, we delineate the hypothetical mechanism of the GH–GHR–IGF1 axis, which may be mostly mediated by IGF1, on the regulation of mitochondrial biogenesis, mitophagy, and mitochondrial function based on the results of studies in the recent decades.

On the other hand, the effects of GH and GHR on mitochondrial function are multifaceted, which may be induced by the differences between cell-, organ-, and species-dependent features or various physiological conditions *in vivo* and *in vitro*. The findings above may lead us to think deeply that GH may exert its multiple effects on mitochondrial function under the direct control by its receptor GHR. However, whether GHR functions as a control valve is still currently lacking in research. Moreover, the molecular basis of GH and GHR on mitophagy is not well understood. In the future, more research is needed to improve our understanding of the effects of GH and GHR on mitochondrial function. This intricate biological process may be explained by the balance between mitochondrial biogenesis and mitophagy under different physiological conditions.

## Author Contributions

BH wrote the manuscript. HL and XZ put forward a lot of invaluable opinions for revision and finalized the manuscript. All authors contributed to the article and approved the submitted version.

## Conflict of Interest

The authors declare that the research was conducted in the absence of any commercial or financial relationships that could be construed as a potential conflict of interest.
